# Simultaneous Clinical Presentation of Renal Cell Carcinoma and Immunoglobulin Light Chain Amyloidosis

**DOI:** 10.7759/cureus.2585

**Published:** 2018-05-06

**Authors:** Bhanu Prasad, Kirsten Tangedal, Rajni Chibbar, Mark McIsaac

**Affiliations:** 1 Department of Nephrology, Regina General Hospital, Regina, CAN; 2 Department of Pharmacology, Regina General Hospital, Regina, CAN; 3 Department of Pathology, University of Saskatchewan, Royal University Hospital, Saskatoon, CAN; 4 College of Medicine, Internal Medicine Training Program, University of Saskatchewan, Royal University Hospital, Saskatoon, CAN

**Keywords:** al amyloidosis, renal cell carcinoma, chronic kidney disease, ascites

## Abstract

A 77-year-old female was admitted to the hospital for an evaluation of congestive heart failure. She gave a history of progressive peripheral edema over eight to 10 months, extending up to the knees bilaterally. Admitting creatinine was 148 mmol/L, serum albumin was 15g/L, and urine protein on quantification was 9.09 g/day. Her immunoglobulin G (IgG) level was 18.4g/L and serum-free kappa level was 92.3 mg/L. The immunofixation of urine revealed monoclonal IgG kappa (1.97 g/d). Her kidney biopsy subsequently confirmed the diagnosis of immunoglobulin light chain (AL) amyloidosis. During the course of investigations, it was incidentally noted that she had a mass on her right kidney, which on biopsy was identified as renal cell carcinoma (RCC). This case deals with the rare situation of AL amyloidosis existing with a solid organ carcinoma and the therapeutic dilemma of treating two unrelated conditions involving the kidneys.

## Introduction

Amyloidosis in the context of RCC usually presents as amyloid A (AA) amyloidosis (systemic amyloidosis) in up to 5% of patients and reflects a chronic inflammatory response, as the amyloid fibrils are composed of fragments of the acute phase reactant serum amyloid A protein [1-2]. This is the first reported case of immunoglobulin light chain AL (kappa) amyloidosis with nephrotic syndrome occurring in conjunction with renal cell carcinoma.

## Case presentation

A 77-year-old Caucasian female was admitted to the hospital for an evaluation of congestive heart failure. She gave a history of progressive peripheral edema over eight months, extending up to the knees bilaterally. She felt weak, exhausted, and had lost her appetite. She denied any orthopnoea or paroxysmal nocturnal dyspnea. She conceded to a long-standing history of hypertension, chronic atrial fibrillation, and hypothyroidism but denied any history of diabetes. Initial investigations, which included a urine analysis, revealed the presence of protein and no evidence of blood. Her admitting creatinine was 148 (micromol/l); serum albumin 15 g/L, and ACR 1025 mg/mmoL (normal <2.8 mg/mmoL). She had a normal white cell count and platelets and her hemoglobin was 115 g/L.

The cause of proteinuria was investigated further and the findings were - serum IgG: 18.4 g/L (5.5-17.24), IgA: 0.51 g/L (0.7-3.94), IgM: 0.53 g/L (0.44-2.47). Serum-free kappa light chains were elevated: 92.3 mg/L (3.3-19.4), and free lambda was 11.7 mg/L (5.7-26.3), kappa/lambda ratio 7.89 (0.26-1.65). b2-microglobulin was 6.8 mg/L (0.0-3.4) and on serum protein electrophoresis (SPEP), there was a presence of an M-spike of 17.2 g/L. Serum immunofixation revealed IgG kappa, and 24-hour urine protein was 9.09 g/day (normal <150 mg/d) with 1.97 g/d of monoclonal IgG kappa. She subsequently underwent a bone marrow biopsy, which revealed 5%-10% small clonal plasma cell population and bone marrow for flow cytometry showed the presence of clonal cell population. The presence of a monoclonal protein and renal impairment raised the possibility of AL amyloidosis, multiple myeloma, immunotactoid glomerulopathy, monoclonal immune deposition disease (light chain disease and heavy chain disease), proliferative glomerulonephritis (GN) with monoclonal deposits, and paraprotein-associated C3 GN.

**Figure 1:**
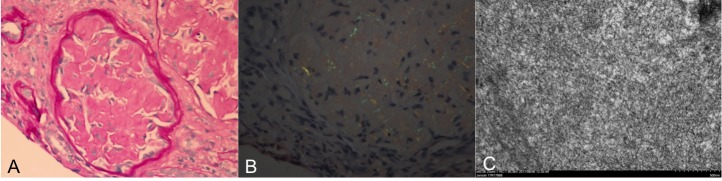
Renal biopsy (light microscopy, immunofluorescence, and electron microscopy) Amyloidosis: a. Glomeruli with mesangial expansion by acellular, pale eosinophilic material (PAS, 400X); b: Congo red stain viewed under polarized light demonstrates apple green birefringence, c. Randomly arranged non-branching fibrils with a diameter of 10-12 nM on electron microscopy.

She subsequently underwent a confirmatory kidney biopsy. On light microscopy, there were 21 glomeruli, four of which were sclerosed. Congo red positive staining was identified. Electron microscopy revealed haphazardly arranged thin fibrils in the mesangium and glomerular basement membrane (Figure 1). Upon further interrogation of the biopsy with liquid chromatography tandem-mass spectrometry (LC MS/MS), a peptide profile consistent with AL (kappa) type amyloid deposit was identified. These changes were histologically consistent with a diagnosis of AL amyloidosis. Clinically, she was diagnosed with AL amyloidosis with nephrotic syndrome.

**Figure 2:**
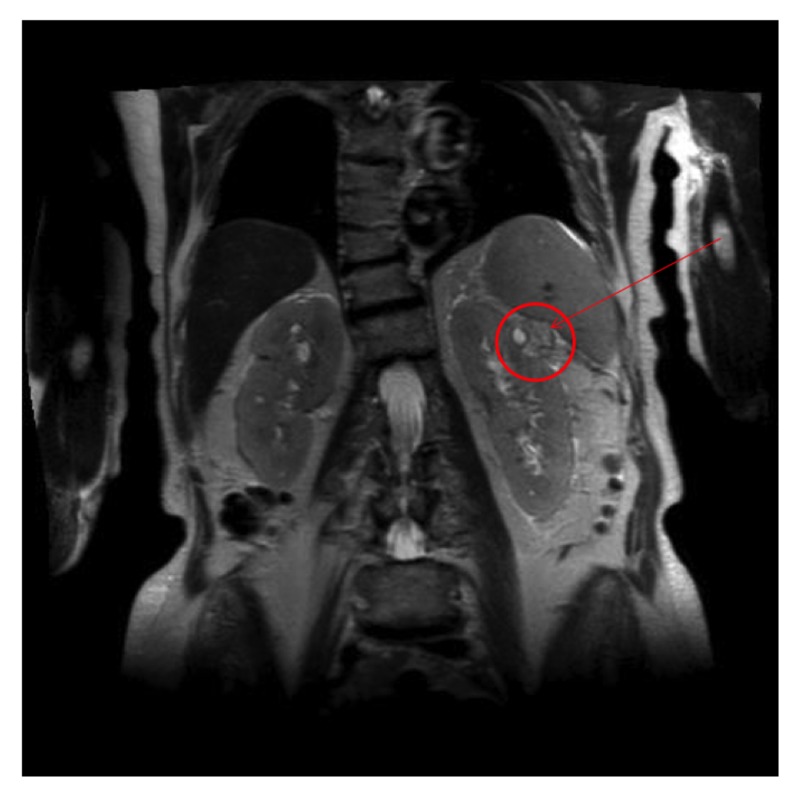
MRI of the kidneys The highlighted renal mass was biopsied and proven to be renal cell carcinoma. MRI: magnetic resonance imaging

During the biopsy, it was noted that there was a solid mass in the left kidney. We investigated it further with computed tomography (CT) of the abdomen, which identified a 2.1 x 1.9 cm cystic lesion (23 Hounsfield units), but in the absence of contrast, the radiologist couldn’t make a further determination. Subsequent magnetic resonance imaging (MRI) revealed a 2.3 X 2.2 cm lesion in the upper pole of the left kidney suspicious of a tumor (Figure 2) and the presence of a single retroperitoneal lymph node, which was biopsied and identified to be benign with no evidence of amyloid. The biopsy of the renal mass was consistent with grade 1 renal cell carcinoma. Her inpatient stay was further complicated by progressive ascites, which required repeated paracentesis. The presence of two concurrent, yet unrelated, diseases involving the kidney led to a therapeutic dilemma. After a discussion with the oncologist, it was decided that RCC was a slow-growing carcinoma and that surgical treatment could be offered upon stabilization of her underlying amyloidosis. She was initiated on 28-day cycles of 1.2 mg/m^2^ of bortezomib, 500 mg of cyclophosphamide, and 20 mg of dexamethasone. Unfortunately, despite the initiation of therapy, her proteinuria worsened, leading to repetitive drainage of her ascites and, later, she became neutropenic and septic. The family later withdrew active care, and she was transitioned to comfort measures and passed away peacefully three days later.

## Discussion

Immunoglobulin light chain (AL) amyloidosis is a protein misfolding disease characterized by the extracellular deposition of an amyloidogenic monoclonal light chain [3]. The amyloidogenic light chains (LCs) are produced by B-cell clones, most frequently a plasma cell. This process results in progressive organ injury that can lead to death in months to years. The kidney along with the heart remains the most common targeted organ. Despite recent advances in pharmacotherapy, both renal and overall prognosis remain poor.

Okamoto et al. recently published a case of AL amyloidosis existing concurrently with lung adenocarcinoma [4]. There are no other recent case reports or series that have documented the association of AL amyloidosis with solid organ tumors. The majority of the association of renal cell carcinoma with amyloid is AA amyloid and reflects a chronic inflammatory response as the amyloid fibrils are composed of fragments of the acute phase reactant serum amyloid A protein. In our patient, the presence of AL amyloidosis and RCC lead to the involvement of multiple care providers and the decision was based on her poor premorbid state of health, frailty, the lack of aggressiveness of the underlying solid organ neoplasm, and intractable ascites as a consequence of hypoalbuminemia from nephrotic syndrome secondary to amyloidosis.

## Conclusions

To our knowledge, this is the first reported case of concurrent RCC with AL amyloidosis. The previous reports in the literature are of AA amyloidosis. The treatment decision depends on the relative aggressiveness of the underlying disease states and the premorbid state of the individual. AL amyloidosis is associated with a high rate of mortality and the outcomes, irrespective of the treatment strategy, remain poor.
